# Small Towns and Land Reform in Zimbabwe

**DOI:** 10.1057/s41287-020-00343-3

**Published:** 2020-12-07

**Authors:** Ian Scoones, Felix Murimbarimba

**Affiliations:** 1 STEPS Centre, Institute of Development Studies, University of Sussex, Falmer, Brighton, BN1 9RE UK; 2 Independent Consultant, Masvingo, Zimbabwe

**Keywords:** Small towns, Land reform, Local economic development, Zimbabwe

## Abstract

Zimbabwe’s land reform from 2000 radically transformed the agrarian structure, and with this small towns in rural areas. This article explores three such towns—Mvurwi, Chatsworth and Maphisa—examining changes in population, housing, transport and business activity between 2000 and 2020. Case studies highlight the importance of networks and social relationships between rural and urban areas, linked to new patterns of migration and a massive growth in the informal economy. Despite the lack of state investment in basic infrastructure, the economies of these small towns have grown significantly, with a major shift in agrarian relations generating new economic activity and employment. This suggests the potential of a territorial focus for local economic development following land reform, encompassing both urban and rural areas.

## Resumé

La reforme rurale du Zimbabwe a, à partir de l’année 2000, radicalement transformé la structure agraire, et avec celle-ci les petites villages dans les zones rurales. Cet article explore trois parmi ces villages - Mvurwi, Chatsworth and Maphisa – en examinant les changements de population, de logement, de transport et d’activité commerciale entre 2000 et 2020. Etudes de cas soulignent l’importance des réseaux et relations sociales entre les zones rurales et urbaines, associés aux nouveaux modèles de migration et à la croissance massive de l’économie parallèle. En dépit de la manque d’investissement publique dans les infrastructures de base, les économies des petites villes étudiés a augmenté de manière considérable, avec un changement important dans les relations agraires, ce qui a généré des nouvelles activités économiques et des nouveaux emplois. Ceci suggère que, suite a des reformes rurales, viser un axe territorial (qui comprend des zones urbaines et rurales) offre du potentiel au développement économique locale.

## Introduction

Following the major land reform in Zimbabwe from 2000, the rural landscape has been transformed (Moyo 2011; Scoones et al. 2010). With a reconfigured agrarian structure the relationships with urban areas have changed too. For three contrasting settings across Zimbabwe, this article asks what difference has land reform made for small towns situated in areas dominated by a restructured agricultural economy?

The literature on small towns and rural–urban linkages in Africa has not addressed the context of land reform. Most discussions point to evolutionary changes as agricultural areas prosper and markets expand, small towns grow with linkages forged through markets, transport and labour exchange generating multiplier effects through a non-farm rural economy (Christiaensen et al. 2013). This is often framed in terms of a ‘structural transformation’ whereby low productivity agriculture is replaced by increasingly industrialised economies (McMillan and Heady 2014), with a concomitant process of deagrariansisation (Bryceson 1996) and growth in urban areas through migration (De Brauw et al. 2014).

Others focus on the spatial relations involved, with the growth of small towns being vital in wider economic change (Dorosh and Thurlow 2013; Pedersen 1997; Simon 1992; Baker 1990; Hardoy and Satterthwaite 1988). This might involve significant infrastructure investment to encourage agriculture-linked industrial activity creating ‘growth poles’ (Picard et al. 2017), ‘corridors’ (Chome et al. 2020) and ‘nodes’ of economic activity (Hinderink and Titus 1988). As rural–urban commerce grows, the importance of supply chains, transport networks, processing facilities and connections to retail outlets are emphasised. Value thus is added to agricultural production in local and regional economies (Berdegué and Proctor 2015; Nel 2005; Rondinelli 1988), although depending on the agricultural value chain, the implications for small towns may differ (Lazaro et al. 2019).

Many have commented on the importance of multi-location and multi-activity households, as people within a household take on different roles on- and off-farm (Steel et al. 2019). This may have a gendered and generational dynamic, with women and younger people engaging in trading and new businesses (Agergaard et al. 2019; Tacoli and Agergaard 2017; Tacoli and Mabala 2010). A movement of people between rural and urban homes, with income-earning activity spread across sites is common (Ingelaere et al. 2017), linked to longer-term patterns of circular migration (Potts 2010).

The changing relationship between the countryside and towns, especially small towns embedded in rural landscapes, often provokes new patterns of accumulation and so processes implications for food security and poverty reduction (Djurfeldt 2015). Some are able to make the most of new market linkages and business opportunities combining increasingly commercialised agricultural production with off-farm income earning, while others cannot (Haggblade et al. 2007). Spatially reconfigured economic activity and associated patterns of class formation, with people straddling urban and rural spaces, is also frequently related to shifts in political relations, prompted through decentralisation policies, for example, or emerging as a result of the role of business elites in small towns linked to rural areas (Owusu 2013; Vincent 1974). Changing relationships between the state and local political authority raises important questions of governance in small towns as new interest groups and power dynamics emerge (Satterthwaite and Tacoli 2003).

**Fig. 1:**
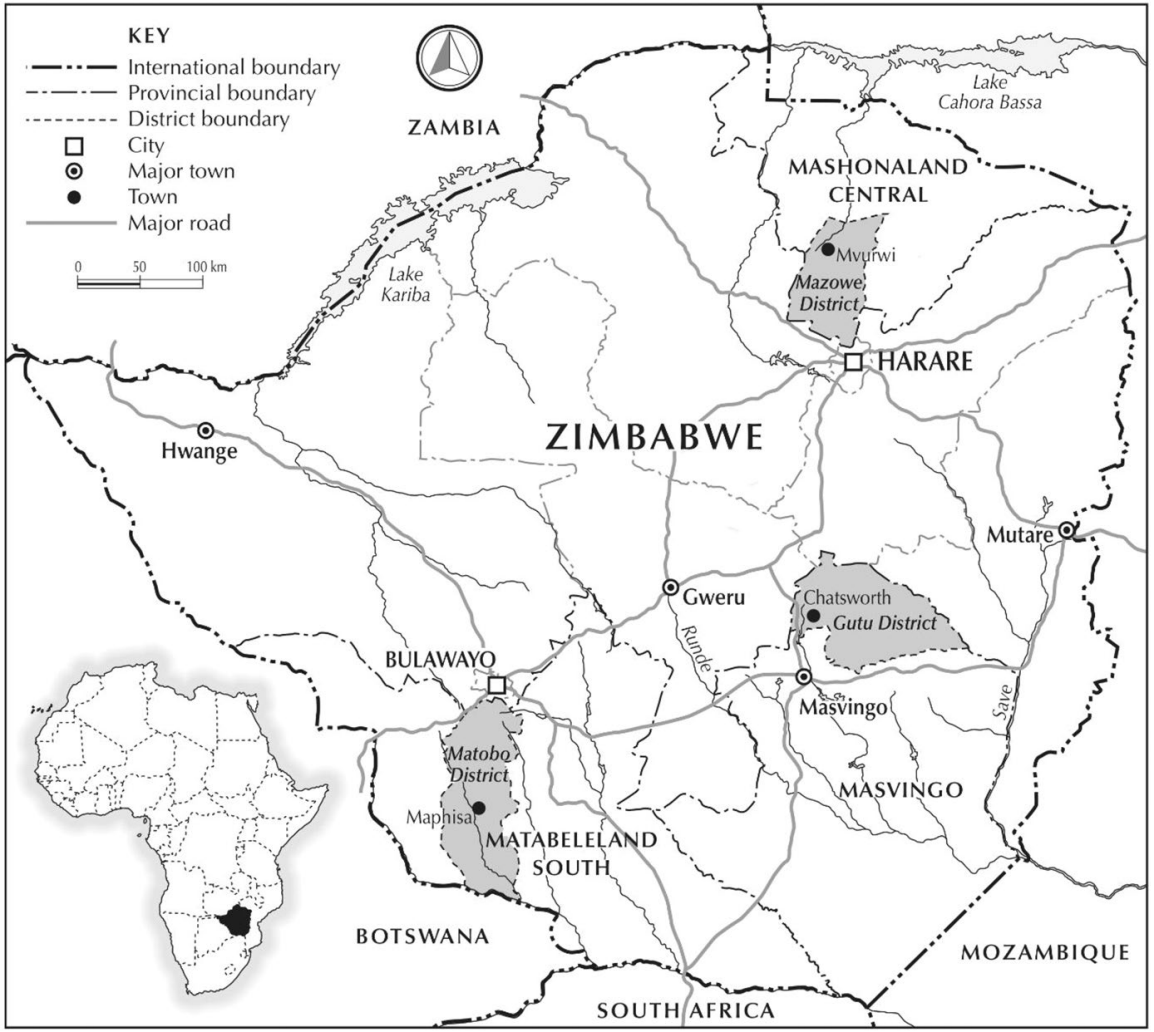
Study areas

Responding to these debates, the article examines three Zimbabwean small towns, each located in areas that have undergone major changes due to land reform since 2000 (Fig. 1). The land reform saw around 9.3 million ha of formerly large-scale commercial farms redistributed to two major resettlement types: A1 (smallholder farms, 145775 farms over 5.8 million ha) and A2 (medium-scale farms, 22896 farms over 3.5 million ha) (Moyo 2011, p. 498). This upset the former dualistic agrarian structure, with large-scale, largely white-owned commercial farms separated from communal areas, where the majority of farmers live. Today there is a more variegated land-use, with smallholder areas (A1 resettlements and communal area farms), medium-scale (A2) farms and remaining large-scale farms and estates sitting alongside each other. The relationships with towns have changed too, with the colonial separation between ‘white’ towns linked to large-scale farming and mining areas and ‘African’ towns and ‘growth points’ serving communal areas being disturbed. Depending on the focus of post-land reform agriculture and its spatial configuration, the impacts on small towns differ, but, as our cases show, an intensification of local economic activity—much of it informal and linked to agricultural production—has dramatically changed the linkages within local economies, and so small towns. The article argues therefore that taking small towns, as linked to wider and now transformed rural areas, seriously is essential if the wider economic benefits of land reform are to be realised.

The article is organised as follows. The next section briefly discusses small towns and urban development in Zimbabwe, locating this in the wider literature on small towns and rural–urban linkages introduced above. Next, the case study areas and the methodology employed is introduced. The results follow in the next section, looking at changes between 2000 and 2020. Four key themes are then drawn out, before the conclusion, which argues for a reframing of the policy debate to focus on the territorial connections around small towns emerging after land reform.

## Small Towns in Zimbabwe: A Brief Overview

Much of the literature on urban settings in Zimbabwe focuses on cities and large towns (e.g. Mbiba 2017; Potts and Mutambirwa 1990), emphasising *inter alia* the role of urban informality (Kamete 2020), the importance of party politics (McGregor and Chatiza 2019; Muchadenyika 2015), the imposition of planning regulations (Vambe 2008) and the livelihoods of workers (Mupedziswa and Gumbo 2001). Many of these themes apply to small towns, but in different ways because of the close connections with the agricultural hinterland.

Where do small towns sit within debates about urban development in Zimbabwe? At Independence in 1980, the new government set about investing in new infrastructure aimed at redressing the imbalances of the colonial past. The 1982 Transitional Development Plan argued for the need to investment in rural service centres: “the intention is to bring the rural population into close contact with services and markets, thus forging linkages with the national economy and stimulating the development of local markets with regional specialisations and a multitude of informal employment opportunities” (GoZ 1982). A hierarchy of urban areas was proposed—consolidated villages, business centres, rural service centres, district service centres, growth points, towns and cities (Mutizwa-Mangiza and Helmsing 1991; Wekwete 1990).

However, in the context of a highly uneven economy, with spatially concentrated, racially defined populations, simply investing in infrastructure and services was not enough. From the colonial period, there were the mining towns, estate towns, white farming towns and TILCOR (Tribal Trust Land Development Corporation) growth points; all had to be incorporated into the post-Independence investment strategy. New district centres and growth points suffered many problems. They were not necessarily integrated into local economies, and without fundamental land reform the dualistic pattern of economic development continued. Some prospered—such as Gokwe thanks to the cotton boom or Murewa due horticultural marketing to Harare—but many remained more in planners’ imaginations than in reality (Wekwete 1988). The lesson of course was clear. For a small town to grow, the agricultural economy around it had to be vibrant and this could not be conjured up by grand plans. It is the wider structural constraints that hold economies back; and in Zimbabwe of course this was substantially to do with access to productive land for the majority.

With some important exceptions (see Andersson 2002; Kamete 1998; Pedersen 1992), however, small towns have not featured significantly in the literature on Zimbabwe. Census data, which defines urban areas as anywhere with a population above 2500 and a majority of non-farm livelihoods, can obscure understanding, as migration between multiple homes is central to mobile, multi-locational livelihoods, with both rural and urban components (Potts 2000a, 1995).

Circular migration between an urban workplace and a rural home was established in the colonial era, as land policies squeezed rural production and forced people (usually younger men) to move in search of work in the large-scale farms, the mines or the growing industrial centres (Arrighi 1970). This pattern has persisted, but as both national and regional economies have changed so have migration patterns (Potts 2000b). Today, however, migration patterns are different, with less predictable movements, especially as the Zimbabwean economy as contracted over the last decades (Crush and Frayne 2010).

Employment in towns and cities is currently limited, particularly for low-skilled jobs, and people have had to seek options further afield. Transnational migration to the UK, US or elsewhere is possible for some (Pasura 2013). Others must face the hazards or illegal migration and casual temporary employment in South Africa; for example in the farms in Limpopo province (Rutherford 2010). With the decline in the formal economy, the pattern of male-dominated migration has shifted, with women increasingly taking up short-term migration for trading (Mutopo 2010). With the arrival of the COVID-19, migration patterns have shifted yet again, making reliance on local forms of income earning even more important.^1^

The extensive literature on migration in southern Africa, and in Zimbabwe in particular, tends to focus on long-distance migration, but frequently ignores shorter movements from rural areas to small towns, even daily commuting. These movements are becoming more important as wider employment opportunities decline and border restrictions intensify. With the growth of agricultural opportunities for some as a result of land reform, there has been a growth in local economic linkages and employment, and a spatial reorganisation of the countryside (Sukume et al. 2015).

Following land reform in 2000, therefore, there were major changes in the rural economy and with these there have been coincident big changes in urban centres—very often small towns—near the new land reform resettlements. To date, this has gone largely un-researched and unnoticed. This article begins to fill this gap.

## Study Area and Methods

To explore these themes, we have explored changes in three small towns near our rural study areas, where we have been examining changes in livelihoods since 2000 among both smallholder (A1) and medium-scale (A2) resettlement farmers (cf. Scoones et al. 2010) (Fig. 1). Like others researching Zimbabwe’s land reform, our attention has not focussed significantly on the wider implications for rural–urban linkages and the changes in small towns near land reform areas.

Our study looked at three such towns. First, Mvurwi in Mazowe district, Mashonaland Central province is around 100 km to the north of the capital Harare. This is the only case that is formally designated a ‘town’ (since 2014) due to its growth in population. Mvurwi was a former service centre for large-scale white farming, as well as being a major farm labour settlement for white commercial farming. Following land reform, it is now at the centre of a booming smallholder-led tobacco growing area. Second is Chatsworth in Gutu district in Masvingo province further south. Chatsworth was once a railway siding, and now is designated as a ‘growth point’. It was in the centre of mostly cattle ranches, but is now surrounded by land reform areas producing maize and vegetables, and is becoming an important marketing and transport hub. Third is Maphisa in Matobo district in Matabeleland South province. Maphisa was formerly a TILCOR growth point, and is again in the centre of a reconfigured rural area, including land reform resettlements and a parastatal farm, now supported through a Joint Venture investment.

Our research involved interviews with residents of all three towns in 2015–16 and 2019, combined with discussions with rural residents in our long-term rural research sites in adjacent land reform areas from 2006 in Masvingo province, 2012 in Mashonaland Central and 2016 in Matabeleland South.^2^ For our urban-focussed research, in total we undertook around 50 interviews across the three sites.

Our aim was to get an overview of the business activities, residence patterns and connections to rural areas from 2000, as well as profiles of investment and accumulation of small town residents. Interviews were complemented with an enterprise survey in each town, enumerating the businesses in operation in three periods. We also compiled data available in local council and government department offices, and referred to statistical data from censuses and other surveys. For insights into longer-term histories, we undertook biographical interviews with long-term residents and consulted archives and past studies to understand the origins and evolution of each settlement.

## Small Towns in Zimbabwe After Land Reform

Table 1 offers an overview of the changes across a selection of indicators for the three towns, based primarily on our enterprise surveys. The data show significant growth in activity since 2000, but variations since.

**Table 1:** Small town profiles, 2000–19

	Mvurwi			Chatsworth			Maphisa		
	2000	2016	2019	2000	2016	2019	2000	2016	2019
Population (approx.)	8100	10500	11000	4500	8380	8500	5400	5500	6000
High-density stands	200	1700	1800	100	300	1580	223	1118	1118
Grocery stores/supermarkets	11	33	63	4	13	21	6	10	12
Market vendors	20	70	120	0	12	12	9	18	45
Butcheries	3	14	8	2	4	3	4	8	8
Hardware stores	4	4	5	1	2	1	1	5	7
Carpentry/welding	5	13	13	1	6	4	2	3	8
Tailors	4	8	8	0	1	2	4	5	3
Food outlets/restaurants	6	20	20	0	2	4	2	5	6
Bars/bottle stores	9	11	10	3	6	5	6	10	8
Hair salons	0	4	4	0	1	3	1	2	4
Hotels/guest houses	2	4	4	0	0	1	3	2	2
Petrol stations	2	6	3	1	1	1	1	2	2
Minibuses/taxis operating	2	35	46	0	15	30	0	30	25

In order to interpret these changes, we have to combine these data with more qualitative insights, as well as data from the nearby rural areas. The following sections introduce each of the small towns in turn.

### Mvurwi: From Farmworker Settlement to Booming Business Centre

On the back of the tobacco boom, Mvurwi town in Mazowe district is a hive of activity, with many new businesses and much new building. Mvurwi (formerly Umvukwes) was formally established after World War II and became an important service centre for white large-scale commercial farmers, as well as a dormitory town for farm workers, including migrants from nearby countries (Scoones et al. 2020).

Before land reform, the central business district of Mvurwi was dominated by farm suppliers, while commercial banks provided credit and tractor hire agencies serviced large-scale farms nearby. Mvurwi commercial farmers enjoyed their leisure time at a country club, built in 1946. Mvurwi has long been a centre for government offices, but the presence of the state has expanded since land reform, with new staff hired. As the town has expanded, more schools have been built and medical facilities expanded.

Since land reform, the agricultural economy of Mvurwi has changed massively. Money from tobacco produced in particular by A1 and A2 farmers has been the driver of Mvurwi’s growth, but in the past, profits were shared among relatively few large-scale white commercial farmers. While workers rented accommodation and would buy basic provisions, they had little disposable income. This all changed following 2000, and particularly after 2009 when the local currency was abandoned due to hyperinflation and the US dollar was adopted. This spurred the expansion of tobacco growing in the area. In our study sites during 2016–17, A1 smallholder farming households generated US$4596 gross income from tobacco and US$2040 from maize on average, while medium-scale A2 farms generated US$60149 and US$30459 gross income from tobacco and maize, respectively.^3^ Farm profits are in turn being reinvested in farms, but also in the town.

Since land reform, there has been a building boom. The town council has allocated many new stands in both high and medium-density areas. Although there have been delays in the provision of basic services (electricity, sewage etc.), these areas have become prime investment sites for farmers, and the town population has expanded rapidly. Our surveys show that 16% of A1 smallholder farmers have invested in building in town since land reform and are receiving rental income.^4^ The massive building projects on-going have generated employment for a range of people including for builders, welders, hardware store owners, brick moulders, sawmill operators and transporters (Table 1). The value of urban land for housing in the town has also attracted corrupt practices, with local politicians becoming involved in selling plots.

Following land reform, a number of businesses focussed on supporting large-scale commercial farming closed as the economy restructured. Yet a multiplicity of enterprises have opened, with expansion reflecting the shift to small- and medium-scale farming in the surrounding areas. For example, stock-feed and day-old chick suppliers have prospered. Butcheries expanded from three to 14 by 2016, with employment growing from six to 30, although several subsequently closed due to electricity shortages. Equally, bars and bottle stores are increasing to service the growing population. General ‘tuck shops’ selling mainly clothes and items from clothing, cell phones, electrical gadgets, to kitchenware have expanded, with about 120 stalls now operating. Currency exchange, eco-cash transfer and mobile phone credit sales have expanded massively too.

Most new businesses are run by locals, very often family members of land reform farmers. Young people and women in particular are important traders. From our surveys in the surrounding A1 resettlement areas, 29% of households are involved in informal trading, while 41% sell vegetables and 23% sell broiler chickens, often in Mvurwi town.^5^ The new, usually temporary, residents of town joined others, including Chinese migrants who have also set up shops and employ locals in their stores. As well as formal businesses, registered with the town council, there have also been a massive shift to the informal economy supporting diverse livelihoods in town.

Such businesses operate at different scales and often interact. For example, informal traders may buy from the Indian-origin-owned wholesale store. Products are then sold on in a range of grocery stores, which have increased six-fold since land reform. MN, a 35-year-old female vendor operating at the open market, explained:

“I buy and sell various farm produce, but my main business is buying and selling sweet potatoes. I buy sweet potatoes from farms and hire scotch carts to bring the produce to the tar road. I manage to buy and sell 36 buckets at US$5 each per week. The business has helped me buy a residential stand and pay school fees”.^6^

Farmers, very often women, from the surrounding areas bring in produce to the town markets, including *masau* berries, indigenous/broiler chickens, cucumbers, tomatoes, rape, *covo* (kale), water melons, cabbages, onions, carrots, green mealies, apples and bananas. Some of these are sold on to food outlets, which have expanded massively since 2000 (Table 1). Mrs G explained how her restaurant is the basis for accumulation:

“I buy Irish potatoes from local farmers which I process into chips served with chicken. I employ a driver who earns US$300 per month as well as six other permanent workers. I have built a crèche, a boarding house for school kids and have plans to build a primary school. I own two private cars and a house in Mvurwi’s low density suburb. I also bought three residential stands”.^7^

Transport business is key for linking farmers to town. As in other sectors, it is increasingly farmers who are investing in assets such as cars and small trucks to transport goods and people. Among our sample of A1 farmers nearby, 20% had bought a car and 18% a truck, while among A2 farmers 41% had cars and 15% had trucks.^8^ Unlike in the period before land reform, the economy is increasingly localised, with benefits being generated in small towns like Mvurwi.

### Chatsworth: From Railway Siding to Growing Small Town

Before land reform, Chatsworth was a cattle loading siding run by Zimbabwe National Railways (ZNR). Surrounded by around a hundred large-scale farms, mostly owned by ‘whites’, it was at the centre of the ranching business. Cattle were loaded onto trucks and taken to the Cold Storage Commission abattoir in Masvingo. Some owned multiple farms over thousands of hectares and managed many thousands of beef cattle. Today, with the exception of one remaining large ranch, all the other farms have been resettled, with a mix of smallholder (A1) and medium-scale (A2) farms.^9^ This has transformed rural production and livelihoods, but it has also transformed Chatsworth.

Before Independence in 1980, Chatsworth was a small outpost with a scattering of shops, some railway employees and a whites-only primary school. There were few businesses, and racial differences were stark. FV recalls: “Greeks and Indians owned the shops in Chatsworth. There was a colour bar. Shops had two entrances: one for blacks and another for whites. Even at the Post Office there were two entrances”.^10^

Chatsworth became more established after 1980. Government offices were set up, and the school grew and allowed all races. But the growth of state presence did not change much in terms of business opportunity. However, after land reform Chatsworth has grown very fast as a rural business and service centre. From a small settlement with 50 location and 50 railway stands, which were home to about 500 people in 2000, residential stands have increased massively and others await servicing (Table 1).

In the past, the railway dominated the town. But by 2019, ZNR employed just two workers in Chatsworth. Prior to the COVID-19 pandemic, the train still ran (erratically) and became an important transport route for women vegetable traders from the areas going to Masvingo to the ‘*kutrain*’ market by the railway tracks. According to Ward councillor, BB:

“Traders board the train to Masvingo town and sell tomatoes, vegetables, green mealies and grain bought at cheaper prices from surrounding land reform farms. They have established relations with farmers sourcing agricultural produce for resale and consumption”.^11^

In our surveys in two nearby A1 resettlement areas, we found that 30% of households earn income from selling vegetables and 10% from selling chickens. Several had also invested in stands and buildings in Chatsworth town since land reform. Indeed, many of the new homes in Chatsworth have been built by those combining trading with agricultural production. Some rent out spare rooms at their Chatsworth residences to tenants who include civil servants. Some female farmer vendors have become part of a new local business elite and invested in transport businesses, owning cars, minibuses and small trucks, while others rent shops.^12^ Civil servants—the formally employed class—do not have houses, as they do not have disposable income to buy stands, and must rent from the new landlords.

Business activity has increased in Chatsworth since land reform. Today farmers on nearby resettled farms and their workers visit Chatsworth each day. In the past, Chatsworth had no supermarket, but today there is one, along with a number of grocery stories, butchers and agro-dealers, each employing people. There are also a number smaller ‘tuck shops’, selling groceries and take-away food, and making a decent profit despite Council fees. By 2019, with the decline in the economy, these businesses had contracted, and with COVID-19 during 2020, some had closed. Hardware stores selling ploughs, harrows and cultivators were faring better. A worker at one of the stores commented: “Business is good. We are selling most building materials to increasing number of people constructing homes in Chatsworth. Resettled farmers are also our clients. The expense of buying from afar forces them to buy from us”.^13^

In addition to the formal stores, there is now an open market selling vegetables. It is operated by local women, based in resettlement areas. One explained: “We order cheaper vegetables from the resettlement farmers for resale but they follow us here and compete with us for customers selling door-to-door to Chatsworth residents and schools”.^14^ With the contraction of the economy and the high costs of operating formally, there has been a shift to more informal trading in Chatsworth, with farmers coming and selling without a market stand. Opportunities in Chatsworth are highly seasonal, with major prayer meetings being an important source of business. Store-owner, Mrs C explained:

“Many churches are active here. The international centre of the AFM (Apostolic Faith Mission) is located here. AFM holds an annual prayer meeting attended by thousands of worshipers from all over the world. There are other numerous churches: Roman Catholic, Dutch Reformed, Zion and others. This creates a big demand for accommodation, but also other business”.^15^

As a small town between Masvingo and Gutu-Mpandawanda, both much larger settlements, businesses in Chatsworth must compete. Ease of transport benefits many but not local businesses. In addition to the train to Masvingo, in 2019, there were multiple minibuses, making access to other towns very easy prior to the COVID-19 movement restrictions.

Chatsworth today—now formally designated a ‘growth point’—is very different to the railway siding supporting the livestock industry of a few large-scale, white-owned farms of the pre-land reform era. As a small urban centre it must compete with larger, more established towns and margins on businesses are small, but in the periods since 2000 when the economy was more stable, there has been significant investment driven by agricultural producers and traders.

### Maphisa: In the Shadow of an Estate

Maphisa (formerly Antelope) was established as a town in the 1970s as part of TILCOR’s attempt to create ‘African towns in African areas’. Mrs N, who was born nearby, explained:

“Maphisa was a forest, a grazing area for livestock. There was an aerodrome for white farmers. A white man called Fish was sent to address the local community to justify building a township. He explained that Antelope dam and irrigation were going to create jobs and benefit communities, who would in turn invest at the township and grow rich! The chiefs and local leadership present at the meeting agreed and Maphisa was established”.^16^

Post Independence these early ‘growth points’ were incorporated into the wider spatial planning approach. The huge estate was taken over by ARDA (the Agriculture and Rural Development Authority), and for several decades Maphisa became intimately linked to the success of the nearby estate, which employed up to 8000 people at the height of the 1990s cotton boom. ARDA also began to build infrastructure in Maphisa in the mid-1980s, including housing for workers and some general dealer shops. The government also established administrative offices for various government departments at the time, and built the Hlalanikuhle high-density location.

Maphisa was surrounded by large-scale commercial ranches, supplying beef to abattoirs in Bulawayo, and many farms also had commercial gold mines on them. The impact of this largely white-owned farming-mining economy on Maphisa was limited, however, as economic linkages were not local. This all changed with land reform, with most farms taken over. In the new resettlements, investment has increased, although mostly from cattle sales rather than crop production. According to 2017–18 surveys,^17^ 70% of medium-scale A2/3-Tier farmers had sold cattle in the previous year, while 28% of A1 farmers had done so. Investments in cars/trucks were significant across the samples, with 72% and 22% of A2/3-Tier and A1 farmers owning cars. Investments in rental accommodation, including in Maphisa, also increased, with 18% and 15% of households involved in A2/3-Tier and A1 areas, respectively.

While land reform areas grew, from 2000 the fortunes of the estate declined, with many laid off. The estate outgrowers (132 families) carried on making use of canal irrigation, but got little support from the estate. The estate’s decline though had a negative effect, as revenues gained from labourers working on the estate vanished. In 2015, a new investment partnership was agreed,^18^ but this new highly mechanised operation has not created the level of employment seen before.

With new people on the land, Maphisa has changed from an estate-linked enclave town to one serving the wider area, with a whole range of new businesses established. As Table 1 shows, there has been considerable growth in the numbers of shops and other businesses in Maphisa since 2000. There are now more people living in the town, and investing in property. Mr T, a local businessman and long-term resident, as well as A2 land reform beneficiary with a 350 hectare farm, explained the impacts of land reform on business:

“Land reform opened up more grazing land and opportunities for livestock marketing. I have 80 cattle, mostly Simenthal crosses. I get around US$800 per beast in Bulawayo. I have just too many goats at the farm! Prices are good: I can get US$50 per goat. The new cattle business is helping Maphisa to grow. For example, hides and skins are available for establishing a tannery industry. Also, there are plenty of mopane worms (*Gonimbrasia belina*) in the farms. Value addition and packaging could be done here”.^19^

Mr N was also born in the area, and has owned shops in Maphisa over many years. He commented on his business: “We sell products to civil servants, ARDA employees, irrigation outgrowers, miners, communal and resettlement farmers and in transit customers. Up to 200 customers cross our doors per day”.^20^ Others rent shops from the council or richer property owners and link to the wider cattle business on the new land reform farms. Mr M explains:

“I buy cattle for US$400–500 on the hoof after bargaining with the seller. I take the beasts to Maphisa council slaughter facilities. I buy 2–3 beasts per week and sell meat to customers at US$5 per kg. My food outlet business is a strategy to increase turn-over of meat sales from the butchery. My wife supervises the business while I run around looking for slaughter stock. I also have an A1 land reform farm with 30 cattle, and these support my business”.^21^

As in the other small towns, the growth of informal trading in Maphisa has been huge. Mrs N is a trader selling vegetables. These were originally supplied by the ARDA estate, but now resettlement farmers supply them: “We use cell phones to communicate and they bring the produce here. Although we have a farm nearby, proceeds from the market have been critical in keeping the home in town going—purchasing food and groceries, paying school fees and council rates”.^22^

Changes in the mining economy have also affected Maphisa. Before, mining was formal and relatively large scale, largely run through the Falcon Gold Company, with worker compounds built on the large-scale farms and with little contact with the wider area. Today this has changed dramatically, with many new mining operations in the area. Mr T, a bar owner, commented: “There are now well over 100 black miners with licences here. Night life is alive due to gold miners”.^23^ Each small mine operation employs around 30 people, all purchasing goods and services in Maphisa.

From an enclave town, linked to an estate, created through colonial, racially based planning, Maphisa has transformed into a business hub linked to local economic activity in both agriculture and mining. The estate remains important, and especially since the injection of new investment, but the town has a more diversified base today. Like the other small towns profiled earlier, Maphisa illustrates the opportunities, but also challenges, of small towns in a restructured economy following land reform.

## Big Changes in Small Towns: Four Themes

Looking across the three cases and the changes observed since land reform in 2000, what have been the major themes emerging? In important respects, the experiences of the three small towns have been different over the past 20 years. Mvurwi’s growth has been driven by the tobacco boom following land reform, while Chatsworth has benefited from the growth of maize and vegetable production, but also the presence of popular churches in the area. Maphisa has grown thanks to the cattle and mining economy, which is now more locally rooted involving many land reform farmers. In all cases, these new economic activities have resulted in important changes in these small towns, although the precise dynamics depends on demographic patterns, the nature of the linked agricultural value chains and the availability of and access to alternative markets. Transformations of rural–urban linkages are therefore highly contingent and context-dependent (Lazaro et al. 2019). In this section, we highlight four cross-cutting themes that characterise the new dynamics of small towns in Zimbabwe’s post-land reform setting across the cases.

### Business Opportunities

The expansion of small- and medium-scale agriculture since land reform has resulted in income from agricultural surpluses being circulated locally. This includes cash from sales of tobacco (Mvurwi), maize/horticulture (Chatsworth) and livestock (Maphisa). In Maphisa, in particular, this agricultural income is complemented by money from small-scale gold mining. This is generating demand for services, as well as opportunities for the sale of produce. Compared to the pre-land reform situation, there are many more businesses of all types in all three towns (Table 1). While some large businesses have closed operations as they were geared towards a different agrarian setting, those that have replaced them are a mix of formal, registered businesses (paying rates and other taxes) and a host of informal businesses. In the past two decades, in part because of the sustained economic crisis that the country has faced, the economy as a whole has informalised. Street vendors, sellers of phone credit, mobile repair operations, transporters and so many more have set up business.

Past patterns of migration—to a stable job in town or in the mines or on the farms—rarely occur today as such jobs no longer exist, and people have to make do in the informal/shadow economy locally. Many of these small businesses are generating employment for others, and so have multiplier effects across the local economy. Across our three cases since land reform, there are five times as many hardware stores, four times as many grocery stores and food outlets, three times as many butcheries and double the number of bottle stores and bars. And there are also new outside investors, including ‘black’ capital, as well as Indian, Chinese and other investors, not seen in these towns before. The number of informal vendors has expanded the most, with on average five times as many as before land reform.

According to our studies in all sites, it is the A1 smallholder resettlements that are in particular driving this local economic dynamism. In aggregate, they both produce the most surplus, distributed across many households, and they also require significant locally sourced inputs—fertilisers, seeds, equipment—and services—notably transport—as part of vibrant farming enterprises. Inputs are supplied in town at agro-dealers and small informal sellers, and in all our case studies, transport has become a vital business linking the town with the wider agricultural hinterland.

### Demography and Difference: New People in Town

The population size and composition of the small towns we studied have changed hugely since land reform. In the past such towns provided accommodation and services (groceries, bars etc.) for workers on the nearby farms and estates (notably Mvurwi and Maphisa) and also provided local input supplies and financial services for the large-scale farmers; although many white commercial farmers sourced more cheaply from further afield. There were government officials posted in all these towns, and there were schools and clinics to support the local population. They were by-and-large small, sleepy locations, with limited activity and few people, organised in the colonial style between high, medium and low-density suburbs, with the latter inhabited by whites, government officials and new black elites.

In the last 20 years, populations have more than tripled; and this is just the permanent population, and not those who commute back and forth from the rural areas. Today there are new people in town. In Mvurwi in particular, former workers in commercial farms no longer have jobs on farms and have had to seek alternatives. Some have left, but many have stayed and become engaged in supporting new forms of local agriculture and become involved in town-based business activities (Scoones et al. 2019a). Women and young people are especially important players in the new informal economy of these small towns. Women, for example, may be married to a man who received land through the land reform and will assist at the family farm, but also will be involved in trading businesses in town. Young people are often without land, missing out on land reform in 2000, but may live at home with their parents and run a business in the nearby town (Scoones et al. 2019b).

Moving small distances, seasonally or daily, is the new pattern, with multi-locational households and multiplex livelihoods the norm. The informal activities that dominate are fragile, informal and risky, but offer a livelihood, and when employing one or two others, can generate in total considerable economic activity. Others have moved to town more permanently, and invested in building houses on newly acquired stands, although usually still retaining a rural base. Government officials may not earn sufficient salary to build, but are the new tenants, while farmers, traders and local elites, with access to patronage funds, are the landlords.

### Rural–Urban Social Relationships and Networks

Land reform has created new rural–urban connections. In the past, the rural town was quite disconnected from the large-scale commercial farming operation, beyond being the source of labour and providing some inputs and services. Today such small towns are intimately linked in a much tighter, more integrated local economy. There are relations from rural to urban (marketing of produce, movement of people) and from urban to rural (supplies of labour, inputs, services and provision of transport).

These linkages are fostered through social relationships, which have to be invested in. Agricultural production, marketing and processing are always embedded in such social relationships and networks. Making sure that a vending business in town thrives requires the vendor to have good relationships with suppliers in the nearby resettlement areas, with transporters to ferry goods to town, with council officials who collect rates and inspect premises and with the police, who invariably are looking for a bribe. Investing in connections and building networks is essential; sometimes through payment, often through building personal relationships through kin, friends and others.

Gaining access to land for a stand in town to build a house may equally require investment in relations, and sometimes all sorts of payments. While the level of politically motivated corruption seen in small, rural towns is not the extent seen in larger towns and cities, it still exists, as the scandal in Mvurwi showed. Surviving in town means playing the game, paying the bribes, dodging the police and sometimes pretending to be a committed party follower.

For many, life in small towns is precarious and risky. With money to be made, there are those ready to exploit and harass informal traders.^24^ Having a rural home nearby to return to is important, and today the rural areas surrounding these towns are now full of farms and people. Keeping close to ‘home’—even if you do not have land—remains crucial, and the ability to be mobile and opportunistic is vital in the face of economic uncertainty, especially for young people.

### Planning, Politics and Governance

There has been a massive expansion of housing in all three towns (Table 1). With the continuity of urban planning regulations from the colonial era, they are still organised into a hierarchy of settlement density, but the occupation of each is less racialised today. Many investors, especially in the fast-expanding high-density suburbs, are land reform farmers and traders in agricultural commodities. Those able to generate surplus from land reform areas are the new landlords, very often with teachers, nurses and other civil servants being the tenants. Patterns of accumulation have shifted, as those with access to land invest in real estate and urban businesses from the profits of farming.

While there has been a building boom across the sites, generating opportunities for brick-makers, hardware suppliers, transporters and builders, the wider public infrastructure of all three small towns is in a poor state. The state has not invested significantly in basic maintenance and expansion of roads, sewage systems or electricity supply in these fast-expanding towns. Even though strict planning restrictions are imposed on new buildings, often involving multiple inspections, the basic provision of services is seriously lacking. The failure of the state (local and national) to provide for basic services and infrastructure in rural and urban areas is a source of deep resentment, especially when high-profile expenditures and rampant corruption are highlighted.^25^ In some cases people have had to invest themselves, for example, improving road access to new homes in town or regrading rural roads to ensure transport plies the route to town.^26^

Perceptions of state neglect feed into a new politics in these areas, where new elites—now prosperous business people and property owners in town, but with links to rural areas via resettlement farms—articulate discontent. They may in turn get co-opted by the ruling party through various deals or may take up opposition politics, with all its attendant dangers. Whatever the outcome, the political dynamics of these areas have shifted dramatically. No longer are such towns the extension of the surrounding white farms, but they are part of a much more contested political milieu driven by new patterns of accumulation and class relations generated by land reform. With a differentiated population of farmers across communal area, A1 and A2 farms, it is those who are ‘accumulating from below’ particularly as a result of land reform that are profiting from new urban connections. This includes especially better-off male A1 farmers, but also women and young people who engage in trade and town-based businesses.

The standard approach to urban administration and governance therefore makes little sense, as people straddle urban and rural areas. Yet local government in Zimbabwe has no capacity to support the new array of demands on services, infrastructure and planning support across town and countryside. The growth of the informal economy, outside state planning controls and local taxation systems, equally makes conventional mechanisms of urban planning and administration ineffective. With new interest groups and associated power relations, the politics of the urban–rural nexus after land reform has become highly contested, with a new rural–urban politics emerging (Scoones 2015). How this translates into reformed administrative and governance arrangements for small towns and associated rural areas remains a major challenge in Zimbabwe, as elsewhere (Satterthwaite and Tacoli 2003). There is, as a result, an urgent need to revive local government’s technical and revenue-raising capacities, alongside reinforcing lines of accountability both with local citizens and the central state for a new post-land reform setting.

## Conclusion: Rethinking Rural–Urban Relations After Land Reform

The role of urban areas in Zimbabwe’s restructured agrarian landscape after land reform has changed. In the past, rural small towns were often situated within large-scale commercial farming areas, where economic linkages were limited. They were dormitory towns for workers and sites for providing limited services.

Today, small towns in rural areas are much more intimately connected to the wider agrarian economy, with more regular movements, closer linkages and tighter social and economic networks binding rural and urban spaces. With greater opportunities for more people to produce agricultural surpluses following land reform—particularly in the smallholder A1 land reform sites—this has meant a rapid growth in economic linkages between rural and urban economies through marketing, labour exchange, service provision and transport.

In the past, the popular imaginary of ‘town’ was somewhere distant, where (mostly) men sought salaried employment as part of circular migration. Now, ‘town’ is closer—physically and psychologically—to rural homes, where men, but also importantly women and young people, can seek off-farm incomes. With lower transport costs driving down local prices, people shuttle between houses in town and on the farms and families are split and mobile; seasonally, but also daily, as there are always full vehicles coming to and from the resettlement farms.

With changing patterns of accumulation, driven in particular by agriculture (and also small-scale mining in Maphisa), there are new patterns of investment and business activity in these small towns. Much business has direct links to agriculture: from local marketing of produce to selling on to restaurants and food sellers to input supplies to equipment sales to financing and transport. Farmers with surplus also need services and consumption goods, and the grocery stores, tailors, hairdressers, bars and restaurants are full, particularly in marketing season. The rise in real estate development and investment among farmers has been an important phenomenon in all sites, but especially in Mvurwi and Chatsworth where surpluses from tobacco and horticulture sales have been invested in bricks and mortar, with many moving into rental businesses to complement farm income.

These opportunities are only available to some of course, but the accumulators include women and young people who have profited from small town-based businesses. However, with the economic decline in Zimbabwe—especially in the mid-2000s and since around 2016, when inflation combined with currency collapse—many businesses have suffered. Indeed, between our enterprise surveys in 2016 and 2019 we saw significant closures of formal businesses, but also an expansion of more informal arrangements. In 2020, with the COVID-19 pandemic and restrictions on opening hours and movement between areas, many businesses closed. While a vibrant local economy linking rural and urban areas and centred on small towns is possible, it is not guaranteed and is highly dependent on the wider macro-economic and political situation.

The new economic configuration emerging following land reform requires rethinking standard planning approaches, which see urban and rural areas as separate. Thinking in terms of local economic development (Nel 2005), and facilitating linkages and multipliers between rural and urban economies (Tacoli 2002) as part of a ‘territorial’ approach (Losch 2016), is essential. As a result, new development priorities emerge. For example, feeder roads to rural areas become important, as does the provision of low-cost and safe market stands and temporary accommodation for mobile traders and business people. Focussing on the changing demographic of urban populations is significant too, with the need to provide safety and security for young and female mobile populations, who are essential to the urban economy often without permanent homes in town. This suggests a recasting of debates about ‘structural transformation’, with a focus on territorial connections across rural and urban spaces (Losch et al. 2012).

As our studies across Zimbabwe have shown, land reform has not only reshaped the agricultural economy, but resulted in major changes in rural small towns. Without much government support, people have refashioned urban and rural spaces and the relationships between them in ways that neither the planning textbooks nor census data reveal. Over the last twenty years, such changes cannot be described in terms of a simple dynamic of African urbanisation (Pieterse and Parnell 2014) (although towns have certainly expanded); nor in terms of processes of ‘deagrarianisation’ (Bryceson 1996) (although some farmers have certainly diversified to non-farm income sources); nor as an outcome of a gradual process of transformation to an urban-industrial economy (McMillan and Headey 2014), as the land reform represented a sudden, radical rupture.

This study therefore complements the now extensive work on livelihoods and rural–urban linkages, adding the implications of land reform to understanding rural–urban dynamics, and especially emphasising the importance of a networked, interlinked local economy, connecting rural and urban spaces through highly contingent and context-specific social, economic and political relationships (Agergaard et al. 2019). A focus on actors, agency and relationships—inflected by social difference—is highlighted that goes beyond simply a structural description of linkages to understanding why and how these are constructed.

Zimbabwe’s land reform has certainly afforded the opportunity for some to accumulate and invest, although not everyone. Others without land can prosper through the income-earning opportunities in urban areas generated by a renewed local agriculture. The result is a variegated pattern in the small town economy that requires a focus on class, gender, age and other dimensions of social difference, in turn with important implications for both rural and urban politics.

In terms of research and development priorities, this post-land reform rural-urban configuration means going beyond a separated town and countryside focus to a wider spatial, territorial perspective, looking at sites of accumulation across rural and urban spaces, and the connections between them, focussing on how social and political relations and governance arrangements are able to support these. Small towns in this sense offer a window onto a new set of economic, social and political relations at the heart of Zimbabwe’s new agrarian landscape, and must be central to territorially focussed, regionally connected local economic development efforts into the future.

## Footnotes

1 https://zimbabweland.wordpress.com/2020/09/27/know-your-epidemic-reflections-from-zimbabwe/.

2 Our A1 sample included 220 households near Mvurwi (Hariana/Ruia); 31 households (Clare/Lonely) near Chatsworth and 67 households near Maphisa (Vimbi/Luna). Our A2 samples (also 3-Tier in Matobo) were 39, 51 and 50 in Mvurwi, Gutu and Matobo.

3 Source, APRA survey, 2016–17.

4 Mvurwi 2014 survey (*N* = 220).

5 APRA 2018–19 survey.

6 Interview Ruia Farm, November 2015.

7 Interview Mvurwi, November 2015.

8 A1 data from APRA survey; A2 data from Shonhe et al. (2020).

9 From our surveys in 2018, on average A1 farms were sold 0.45 tonnes of maize (at US$390 per tonne), while A2 farms sold 3.7 tonnes, and 63% of A2 farmers and 27% of A1 farmers sold cattle (at around US$500 per beast) (see https://zimbabweland.wordpress.com/2020/05/11/zimbabwes-land-reform-areas-twenty-years-on-1-introducing-a-short-blog-series/ and Shonhe et al. 2020).

10 Interview, Chatsworth, October 2015.

11 Interview, Chatsworth, October 2015.

12 In our nearby A1 sample, 37% of households owned cars by 2019, while among A2 farmers 45% owned cars and 35% owned small trucks (see https://zimbabweland.wordpress.com/2020/05/11/zimbabwes-land-reform-areas-twenty-years-on-1-introducing-a-short-blog-series/).

13 Interview, Chatsworth, November 2015.

14 Interview, Chatsworth, December 2019.

15 Interview, Chatsworth, December 2015.

16 Interview, Maphisa, May 2016.

17 Tapiwa Chatikobo, pers. comm. (August 2020).

18 See: https://zimbabweland.wordpress.com/2016/10/31/are-joint-ventures-with-parastatals-the-route-to-reviving-large-scale-commercial-agriculture-in-zimbabwe/.

19 Interview, Maphisa, May 2016.

20 Interview, Maphisa, May 2016.

21 Interview, Maphisa, May 2016.

22 Interview, Maphisa, May 2016.

23 Interview, Maphisa, May 2016.

24 See: https://africanarguments.org/2020/03/27/surviving-covid-19-fragility-resilience-and-inequality-in-zimbabwe/.

25 See: https://nehandaradio.com/2020/07/15/vp-mohadi-commissions-us18-million-bridge-size-and-amount-causes-outrage/.

26 See: https://zimbabweland.wordpress.com/2015/02/16/regalvanising-the-state-from-below-how-a-road-got-regraded/.
